# Modified Delphi study to identify priority clinical questions for the Australian living guidelines for the management of Juvenile Idiopathic Arthritis

**DOI:** 10.1186/s12969-022-00710-w

**Published:** 2022-07-23

**Authors:** Georgina Tiller, William D. Renton, Joachim Tan, Samuel Whittle, Jodie Avery, Jane Munro, Rachelle Buchbinder

**Affiliations:** 1 Department of Epidemiology and Preventive Medicine, School of Public Health and Preventive Medicine, Monash University, Melbourne, Australia; 2 Monash-Cabrini Department of Musculoskeletal Health and Clinical Epidemiology, Cabrini Health, Melbourne, Australia; 3 Rheumatology Unit, Department of General Medicine, The Royal Children’s Hospital, Melbourne, Australia; 4 Rheumatology Unit, Women’s and Children’s Hospital, Adelaide, Australia; 5 Rheumatology Unit, The Queen Elizabeth Hospital, Adelaide, Australia; 6 Molecular Immunity Group, Murdoch Children’s Research Institute, Melbourne, Australia

**Keywords:** Rheumatology, Arthritis, Juvenile, Research Priorities, Guideline, Australia

## Abstract

**Background:**

Juvenile Idiopathic Arthritis (JIA) is the most common rheumatic inflammatory disease in childhood. Optimal management requires clinicians to be up to date with the rapidly evolving evidence base. ‘Living’ evidence-based clinical practice guidelines, which integrate new evidence as soon as it is available, are a novel method to enhance the translation of research into practice. To determine the most relevant questions that should be prioritised in national Australian JIA living guidelines, we invited Australian and New Zealand paediatric rheumatologists and other relevant health professionals to identify and rank their most important questions in order of priority.

**Methods:**

All 47 members of the Australian Paediatric Rheumatology Group (APRG) were invited to participate in a modified Delphi study comprising two rounds. The first round identified demographic information of respondents, current attitudes to guideline use and invited submission of priority management questions. The second round asked respondents to rank 27 collated and refined questions identified in round one in order of priority.

**Results:**

There were 29 (62%) and 28 (60%) responses to the first and second survey rounds respectively. About two thirds were rheumatologists or trainees (66, 68%), nearly half had more than 10 years of experience (45, 46%) and practice setting was largely hospital (79, 86%) and urban (86, 75%). Most respondents used clinical guidelines in their practice (72% sometimes, 24% often), most frequently American College of Rheumatology (ACR) (66%) and European Alliance of Associations for Rheumatology (EULAR) (59%) guidelines. Reported barriers to guideline use included that they are not up to date and access difficulties. Most respondents (83%) considered Australian guidelines were necessary and two-thirds indicated they would use them if integrated into practice software. The highest ranked topics were down-titration and discontinuation of disease modifying anti-rheumatic drugs (ranked first), best outcome measures (second) and treatment targets in JIA (third).

**Conclusions:**

There is strong clinician support for the development of Australian living guidelines for JIA. Consensus was reached on the ten top-ranked priority questions. Our guidelines will develop evidence-based recommendations for these high priority questions that will be updated in real time as needed to facilitate rapid translation of evidence into clinical practice.

**Supplementary Information:**

The online version contains supplementary material available at 10.1186/s12969-022-00710-w.

## Background

To effectively practice evidence-based medicine, clinicians need to keep abreast of the latest evidence in their field, an increasing challenge as the volume of new evidence rapidly expands [1]. Systematic reviews are a valuable tool for synthesising evidence but are only useful if they are up to date. ‘Living’ systematic reviews are a new approach that use novel methods such as regular automated searches to continuously update reviews as new evidence becomes available [2].

Living reviews can also be incorporated into ‘living’ clinical practice guidelines by updating individual recommendations within a dynamic, web-hosted guideline structure as soon as relevant new evidence emerges [3]. A further advantage of living guidelines is that they can be developed in a stepwise format, in which additional living recommendations can be added over time as resources permit.

To optimise immediate effect, topics for inclusion can be prioritised using a systematic approach. Such a framework has been used by the 3e (Evidence, Expertise, Exchange) Initiative that has developed international clinical practice guidelines on topics including undifferentiated peripheral inflammatory arthritis [4], pain management in inflammatory arthritis [5] and gout [6]. Using this method, guidance on the most relevant clinical questions was developed.

To our knowledge, this process has not been performed in the field of paediatric rheumatology. Juvenile idiopathic arthritis (JIA) is the most common chronic inflammatory rheumatic disease in childhood and could be well suited to a living guideline format given the expanding range of available treatments. The most recent Australian guideline for the management of JIA was published in 2009 [7] and many of its recommendations are outdated. The Australia and New Zealand Musculoskeletal (ANZMUSC) Clinical Trials Network, in partnership with the Australian Paediatric Rheumatology Group (APRG) and Australian Rheumatology Association (ARA), is developing Australian living guidelines for the management of JIA in parallel with the development of living guidelines for adult rheumatic diseases [8].

Our process of living guideline development provides a unique training opportunity to members of the multidisciplinary guideline panels in evidence-based practice, living evidence and guideline development methods. Involvement of clinicians early in guideline development also improves the relevance of the recommendations and may increase guideline uptake to hasten translation of evidence into practice [5, 9]. To facilitate a living guideline that is immediately useful, we performed a modified Delphi study to reach consensus on the most clinically relevant questions, ranked in order of priority, that APRG clinicians would like addressed in the Australian JIA living guidelines.

## Methods

### Design and participants

We used a modified Delphi technique including two survey rounds [10]. The invitation to the first and second surveys were sent 31 January and 9 March 2021 respectively.

All members of the APRG (*N* = 47) were invited to participate by email. This group includes all currently practising paediatric rheumatologists in Australia and New Zealand, all advanced trainees in paediatric rheumatology, adult rheumatologists with an interest in paediatric rheumatology, and rheumatology health professionals, including physiotherapists, occupational therapists, research assistants and nurses.

To maximise participation, a follow-up email was sent 2 weeks after the initial invitation for each Delphi round. Each survey link was closed 6 weeks after the first email. Individual responses were anonymised. All APRG members were invited to participate in the second round, regardless of their participation status in the first round.

### Survey tool and procedure

An online platform (SurveyMonkey, Momentive Inc. San Mateo, California, USA) was used to collect the data. Surveys from the first and second rounds are appended (Additional files 1 and 2 respectively).

The first round included an introduction to the concept of living guidelines, an overview of the ANZMUSC JIA living guidelines project and described the aim of the study. Participants were invited to submit at least 3 and up to 10 questions they thought were most relevant to the management of JIA via free-text entry. Example questions were provided for guidance. We also collected demographic information and asked respondents about current use of JIA guidelines and beliefs about the need for local guidelines (Additional file 1).

The complete list of questions identified in the first round was refined and duplicate questions were removed. The refined list of questions was provided to respondents in random order in the second round. Respondents were asked to indicate the ten questions they considered most important and then to rank them from most to least important.

### Analysis

Using results from the second round, each question was assigned a score with 10 points for questions that were ranked first to 1 point for questions that were ranked tenth. A priority list was created according to the sum of ranking scores. The final ranked order of questions, their score, and the absolute number of respondents who voted for a particular question are reported.

## Results

There were 29 (62%) and 28 (60%) responses to the first and second rounds respectively. The characteristics of respondents in comparison to the APRG membership as a whole is summarised in Table 1. The gender and discipline breakdown of respondents reflected that of the APRG membership. Most respondents in both rounds were paediatric rheumatologists (first round *n* = 15, 52%; second round *n* = 16, 57%) or trainees (*n* = 4, 14%; *n* = 3, 11%). Thirteen respondents in both survey rounds (45 and 46%) had more than 10 years’ experience in paediatric rheumatology, and the majority work in urban (*n* = 25, 86%; *n* = 21, 75%) and hospital (*n* = 23, 79%; *n* = 24, 86%) settings.

**Table 1:** Characteristics of respondents and their guideline use

	Survey 1 *N* = 29	Survey 2 *N* = 28	All APRG members *N* = 47
	n (%)	n (%)	n (%)
Female	19 (66)	18 (64)	33 (70)
Discipline			
- Rheumatologist	15 (52)	16 (57)	24 (51)
- Rheumatology trainee	4 (14)	3 (11)	6 (13)
- Nurse	2 (7)	1 (4)	3 (6)
- Allied health/ other	8 (28)	8 (29)	14 (30)
Years involved in rheumatology			
- 0–5 years	7 (24)	6 (21)	
- 6–10 years	9 (31)	9 (32)	
- > 10 years	13 (45)	13 (46)	
Primary place of practice			
- Hospital (public)	23 (79)	24 (86)	
- Private practice	0 (0)	0 (0)	
- Combination of public and private	4 (14)	3 (11)	
Location of practice			
- Urban	25 (86)	21 (75)	
- Rural	0 (0)	1 (4)	
- Both urban and regional	3 (10)	5 (17)	
Use of guidelines in usual practice			
- Never	0 (0)		
- Sometimes	21 (72)		
- Often	7 (24)		
Current guideline use			
- CARRA	7 (24)		
- EULAR	17 (59)		
- SHARE	7 (24)		
- ACR	19 (66)		
- GKJR	0 (0)		
- Other	5 (17)		
Reasons for not using guidelines			
- Personal preference	3 (10)		
- Not representative	8 (28)		
- Interrupt clinical interaction	5 (17)		
- Difficult to access	10 (34)		
- Unnecessary with experience	2 (41)		
- Not up to date	13 (45)		
- None of the above	2 (7)		
Are Australian JIA guidelines necessary?			
- Yes	24 (83)		
- No	1 (3)		
- Unsure	3 (10)		

*ACR* American College of Rheumatology, *APRG* Australian Paediatric Rheumatology Group, *CARRA* Childhood Arthritis and Rheumatology Research Alliance, *EULAR* European Alliance of Associations for Rheumatology, *GKJR* German Society for Paediatric Rheumatology, *JIA* Juvenile Idiopathic Arthritis, *SHARE* Single Hub and Access point for paediatric Rheumatology in Europe

Most respondents indicated they sometimes use guidelines in their usual practice (n = 21, 72%), while almost a quarter (*n* = 7, 24%) indicated they used them often. The guidelines most commonly used by respondents were from the American College of Rheumatology (ACR) (*n* = 19, 66%) and European Alliance of Associations for Rheumatology (EULAR) (*n* = 17, 59%).

Several barriers to using existing guidelines were reported. Thirteen respondents (45%) indicated guidelines were not up to date and 10 (34%) indicated they had difficulties accessing a guideline. Most respondents (n = 24, 83%), indicated that Australian paediatric rheumatology guidelines are necessary and 19 (66%) would use them if integrated into practice software.

There were 134 questions proposed in the first round. The most common questions related to tapering of disease-modifying antirheumatic drugs (DMARDs) identified by 12 (41%) respondents, the most appropriate outcomes to measure in clinical practice identified by seven (24%) respondents and immunisations identified by six (21%) respondents. After refinement and duplicate removal, there were 27 questions presented in the second round.

Table 2 shows the most important questions and their rankings as identified in round two. The highest ranked question concerned tapering and discontinuation of DMARDs in patients with JIA who have responded well to treatment (score = 140). It was ranked in the top 10 by 21 of 24 (87%) respondents. Other highly ranked issues related to outcome measures / treatment targets (score = 90, 11 respondents), use of oral glucocorticoids (score = 81, 14 respondents), use of methotrexate (score = 78, 13 respondents), and DMARD strategy based on JIA subtype (score = 78, 12 respondents).

**Table 2:** Top important questions, ranked score and number of respondents who voted for each question in Round 2 (*N* = 24)

Rank	Rank Score	Question	n (%)
1	140	When and how should cs/b/tsDMARDs be tapered or discontinued in patients with JIA who have responded well to treatment?	21 (87)
2	90	What are the best outcome measures and the treatment targets in JIA?	11 (45)
3	81	What is the best approach to the use of oral glucocorticoids in patients with JIA, including weaning strategies and monitoring of side-effects?	14 (58)
=4	78	What is the best approach to using methotrexate in JIA including side-effects, route of administration and screening pre-commencement?	13 (54)
=4	78	What is the best approach to choosing a DMARD treatment strategy based on JIA subtype?	12 (50)
6	74	What is the best approach to choosing a bDMARD in the management of JIA in patients who have not responded to or are intolerant to csDMARDs?	13 (54)
7	71	What is the best approach to choosing a bDMARD or tsDMARD in patients with JIA associated uveitis who have not responded to methotrexate?	11 (45)
=8	51	What is the risk of flare of disease after ceasing csDMARDS, bDMARDS or tsDMARDS for JIA?	11 (45)
=8	51	What is the best approach to the use of steroid joint injections in JIA?	8 (33)
10	48	What is the best DMARD choice in patients with polyarticular JIA who have failed to respond to a first bDMARD?	8 (33)
11	46	What is the best approach to the assessment and management of persistent or amplified pain in patients with JIA?	13 (54)
12	40	What is the role of exercise in the management of JIA?	9 (38)
=13	39	What is the role of methotrexate for the prevention of anti-drug antibodies in patients with JIA using bDMARDs?	10 (42)
=13	39	How should we define remission in JIA?	7 (29)
=13	39	What is the role for non-methotrexate csDMARDS in the management of JIA?	6 (25)
=13	39	Which investigations should be performed before commencing csDMARDS, bDMARDs and tsDMARDS in JIA?	7 (29)
17	38	What is the best approach to the measurement of drug levels and the detection of antibodies in the management of JIA?	9 (38)
18	36	What is the role of imaging in aiding management decisions in JIA?	5 (21)
=19	34	What is the best approach to TMJ steroid injection as a therapy for TMJ arthritis in JIA patients?	10 (42)
=19	34	What is the best approach to the management of varicella screening, immunisation and exposure in JIA?	8 (33)
=21	28	What is the best approach to monitoring patients for the side effects and toxicity of csDMARDS, bDMARDS and tsDMARDS?	4 (17)
=21	28	Which vaccinations should be offered to patients receiving treatment for JIA and when?	8 (33)
23	25	What is the best approach to screening for JIA associated uveitis?	7 (29)
24	20	What is the best initial DMARD treatment in patients with JIA who have not previously received DMARDS?	3 (13)
25	13	What is the optimal frequency for reviewing patients with JIA who are in remission off medication?	4 (17)
26	9	How should anti TNF-α therapy be used in patients with JIA who have a family history of MS?	5 (21)
27	7	What is the role of complementary medicine in the management of JIA?	3 (13)

*bDMARD* biologic DMARD, *csDMARD* conventional synthetic DMARD, *DMARD* disease modifying anti-rheumatic drug, *JIA* juvenile idiopathic arthritis, *MS* multiple sclerosis, *TNF-α* tumour necrosis factor alpha, *TMJ* temporomandibular joint, *tsDMARD* targeted synthetic DMARD

## Discussion

Most health professionals involved in the specialist care of Australian children with JIA and who responded to our survey use guidelines as part of routine clinical care. They were also strongly supportive of locally produced Australian JIA guidelines despite the existence of guidelines for JIA developed elsewhere. Australian living guidelines would ensure recommendations are relevant to the local context. In addition, respondents reported concerns about guidelines often being out of date. A single repository for JIA management recommendations, that are updated as new evidence emerges, and available and accessible at the point of care, would address these concerns.

It has been noted that needs raised by clinicians and consumers are not always reflected in the research that is performed [11]. Topics without clear evidence to guide practice were understandably highly prioritised as being important to respondents. For example, there is a paucity of high quality research investigating down-titration and discontinuation of DMARDs in JIA patients with inactive disease and a large variation in clinical practice currently exists [12]. While recently published guidelines have not addressed this issue, this was our top ranked priority. It has also been identified as a priority topic for other guidelines [13, 14].

The second ranked question, concerning the best outcome measures and treatment targets in JIA, is also not addressed in existing guidelines. Incorporating standardised outcome measures into JIA guidelines would support a ‘treat to target’ approach which an international taskforce has determined to be the accepted strategy in the management of JIA [15]. The taskforce did not specify which instrument should be used to measure disease activity, instead leaving this decision to individual clinicians. Further research is required to address such questions.

While some of the priority questions identified would be applicable to both adult and childhood onset inflammatory arthritis, others are more particular to paediatrics. The management of JIA-associated temporomandibular joint arthritis, for example, is notoriously difficult and evidence-based treatment guidelines are limited [16]. Drug monitoring using antibodies is also a growing area of interest in the field of paediatric rheumatology and is largely not addressed in current guidelines [17].

A recent Dutch nationwide survey of paediatric rheumatology clinicians yielded similar results to our study [11]. This group identified the management of pain and fatigue, medication tapering, uveitis management, individualised treatment and patient self-management skills as high priority topics for clinicians.

There are several strengths to our study. The Delphi method is a robust way of reaching consensus without the agenda being dominated by opinion leaders [18]. The response rates to the first (46%) and second (44%) rounds compare well to other similar surveys [9, 19, 20] and respondents appeared representative of the APRG as a whole based upon the limited variables we could assess. The diversity of experience and professional disciplines among respondents suggests that guidelines will be broadly relevant to health care professionals involved in the multidisciplinary management of JIA. The questions identified in this study pertain to an Australian context however the findings are likely to be generalisable and useful in other similar settings. This study outlines an efficient way to prioritise clinical questions and could easily be replicated in another area of paediatric rheumatology or different healthcare settings. Limitations include the lack of inclusion of other stakeholders including consumers, industry representatives and policy makers. As observed in other studies, inclusion of these groups may affect these priorities [21]. Given the anonymous nature of the data collection, we were unable to determine if there were important differences in clinician priorities based on practice settings, be that urban or regional.

Through a Delphi method we have derived clearly defined questions that clinicians who manage JIA would like answered as part of a living JIA guideline. Since the establishment of this list, an ANZMUSC JIA living guideline multidisciplinary panel comprising representatives from medical, nursing, allied health and consumer backgrounds have made their first living guideline recommendation addressing a question in the top ranked list [22]. The panel made a conditional recommendation for the use of adalimumab for patients with JIA-associated uveitis who have not responded to methotrexate and the findings have been published in an open access format (available at https://app.magicapp.org/#/guideline/5847). Evidence syntheses to inform further recommendations addressing other priority questions are in progress and relevant recommendations will be maintained in living mode and updated over time as new evidence emerges.

## Conclusions

There is strong support among APRG members for the development of Australian living guidelines for JIA and our study has identified the most important clinical questions from a clinician perspective. The results will be used to inform development of our guidelines, and these will be updated as new evidence emerges. This world-first living guideline for JIA represents a significant advance in guideline development and optimisation of care and outcomes of people living with JIA.

## Supplementary Information

**Additional file 1.** First Survey. Description of data: First round survey as provided to participants.

**Additional file 2.** Second Survey. Second round survey as provided to participants.

## Data Availability

The datasets generated during and/or analysed during the current study are available from the corresponding author on reasonable request.

## Abbreviations

ACR: American College of Rheumatology
ANZMUSC: Australia and New Zealand Musculoskeletal Trials Network
APRG: Australian Paediatric Rheumatology Group
CARRA: Childhood Arthritis and Rheumatology Research Alliance
DMARD: Disease Modifying Anti-Rheumatic Drug
EULAR: European Alliance of Associations for Rheumatology
GKJR: German Society for Paediatric Rheumatology
JIA: Juvenile Idiopathic Arthritis
RACGP: Royal Australian College of General Practitioners
SHARE: Single Hub and Access point for paediatric Rheumatology in Europe
