# Supplementary material for: Modified Delphi study to identify priority clinical questions for the Australian living guidelines for the management of Juvenile Idiopathic Arthritis
Source: Pediatr Rheumatol Online J. 2022 Jul 23;20:52. doi: 10.1186/s12969-022-00710-w (PMC9308308; doi:10.1186/s12969-022-00710-w)
Supplement: Supplementary file 1 — Additional file 1. First Survey. Description of data: First round survey as provided to participants. [file 12969_2022_710_MOESM1_ESM.docx]

The Australia and New Zealand Musculoskeletal (ANZMUSC) Clinical Trials Network is developing a set of Australian guidelines for the management of juvenile idiopathic arthritis.

These will be ‘living’ guidelines, in which individual recommendations are updated in near real-time as new evidence emerges. Initially, a relatively small set of recommendations will be developed, but the living guideline format permits further recommendations to be added over time as resources permit.

In order to identify the topics of most importance to potential users of this guideline, we invite APRG members to participate in a prioritisation exercise. In this, the first round, we ask you to nominate at least 3 questions that you think ought to be addressed in a guideline for the management of juvenile idiopathic arthritis.

A second and final survey round will ask participants to help rank the aggregated results of the current survey.

The survey will take less than 10 minutes to complete.

All responses are completely anonymous. Completion of the survey will imply that you consent to participate. You are free to withdraw at any time during the survey and none of your data will be collected.

The study has been approved by the Central Adelaide Local Health Network Human Research Ethics Committee. If you wish to speak to someone not directly involved in the study about your rights as a volunteer, or about the conduct of the study, you may also contact the CALHN HREC Chairperson, on 7117 2229 or 8222 6841.

CALHN reference number: 12728

1. What is your current role?
   1. Paediatric rheumatologist
   2. Adult rheumatologist
   3. Paediatrician
   4. Rheumatology advanced trainee
   5. Rheumatology nurse
   6. Other rheumatology health professional
   7. Other (please specify)
2. Gender
   1. Female
   2. Male
   3. I’d rather not say
3. What is your primary place of practice?
   1. Hospital
   2. Private practice
   3. Combination of public and private practice
   4. Not applicable
   5. Other (please specify)
4. What is your primary location of practice?
   1. Urban
   2. Rural/regional
   3. Both urban and regional
   4. Not applicable
5. Years involved in rheumatology
   1. 0-5
   2. 6-10
   3. 11-20
   4. >20
6. Do you use an electronic health record in your usual practice?
   1. Yes
   2. No
   3. Unsure
7. Do you believe that Australian paediatric rheumatology guidelines are necessary
   1. Yes
   2. No
   3. Unsure
8. Do you use any rheumatology guidelines in your usual practice?
   1. Never
   2. Sometimes
   3. Often
9. What prevents you from using guidelines? (select all that apply)
   1. Personal preference
   2. Difficult to access
   3. Not representative of my patients
   4. Unnecessary because i have sufficient expertise
   5. Interfere with practitioner autonomy
   6. Guidelines are often not up to date
   7. Interrupt with clinical interaction
   8. None of the above
   9. Other (please specify)
10. Would you use a guidelines if it were integrated into your practice software?
    1. Yes
    2. No
    3. Unsure
11. Which JIA guidelines do you use most commonly? (select all that apply)
    1. Childhood Arthritis and Rheumatology Alliance (CARRA)
    2. European League Against Rheumatism (EULAR)
    3. Single Hub and Access point for paediatric Rheumatology in Europe (SHARE)
    4. American College of Rheumatology (ACR)
    5. German Society for Paediatric Rheumatology (GKJR)
    6. Other (please specify)
12. Nominate at least 3 important questions for a guidelines for the pharmacological management of juvenile idiopathic arthritis. You may submit up to 10 questions. Please try to choose brief questions that relate to a specific aspect of your daily clinical practice, for example: “When should I taper DMARDs in JIA patients in remission?” or “What are the best outcome measures in JIA?” or “What vaccinations should be offered to a patient with JIA and when?”
