# Supplementary material for: Modified Delphi study to identify priority clinical questions for the Australian living guidelines for the management of Juvenile Idiopathic Arthritis
Source: Pediatr Rheumatol Online J. 2022 Jul 23;20:52. doi: 10.1186/s12969-022-00710-w (PMC9308308; doi:10.1186/s12969-022-00710-w)
Supplement: Supplementary file 2 — Additional file 2. Second Survey. Second round survey as provided to participants. [file 12969_2022_710_MOESM2_ESM.docx]

Choose the TEN most important questions for a living guideline for the pharmacological management of juvenile idiopathic arthritis.

Please select exactly 10 questions. In the next section, you will be asked to rank your 10 choices in order of importance.

What is the best approach to the use of methotrexate in JIA including managing side effects, route of administration and screening pre commencement?

What is the best initial DMARD treatment in patients with JIA who have not previously received DMARDs?

What is the best approach to the use of steroid joint injections in JIA?

What is the role of imaging in aiding management decisions in JIA?

What is the best approach to the assessment and management of persistent or amplified pain in patients with JIA?

What is the optimal frequency for reviewing patients with JIA who are in remission off medication?

What is the role of complementary or alternative medicine in the management of JIA?

How should anti TNF therapy be used in patients with JIA who have a family history of multiple sclerosis?

What is the best approach to choosing a bDMARD or tsDMARD in patients with JIA associated uveitis who have not responded to methotrexate?

What is the best approach to TMJ steroid injection as a therapy for disease of the TMJ in JIA patients?

After ceasing csDMARDs, bDMARDs or tsDMARDs in JIA, what is the risk of flare of the disease?

What are the best outcome measures and treatment target measures in JIA?

What is the best approach to the use of oral glucocorticoids in patients with JIA, including weaning strategies and monitoring for side effects?

When and how should csDMARDs, bDMARDs and tsDMARDs be tapered or discontinued in patients with JIA who have responded well to treatment?

Which investigations should be performed before commencing csDMARDs or b/tsDMARDs in JIA?

Which vaccinations should be offered to patients receiving treatment for JIA, and when?

What is the best approach to the management of varicella screening, immunisation and exposure in JIA?

What is the role of exercise in the management of JIA?

What is the best DMARD choice in patients with polyarticular JIA who have failed to respond to a first bDMARD?

How should we define remission in JIA?

What is the best approach to choosing a bDMARD in the management of JIA in patients who have not responded or are intolerant of csDMARDs?

What is the best approach to the use of methotrexate for the prevention of anti-drug antibodies in patients with JIA?

What is the best approach to the measurement of drug levels and the detection of antibodies in the management of JIA?

What is the best approach to screening for JIA associated uveitis?

What is the best approach to monitoring patients for the side effects and toxicity of csDMARDs, bDMARDs or tsDMARDs?

What is the role for non-methotrexate csDMARDs in the management of JIA?

What is the best approach to choosing a DMARD treatment strategy based on the JIA subtype?
